# Intensity of Free Radical Processes in Rat Liver under Type 2 Diabetes and Introduction of Epifamin

**Published:** 2013

**Authors:** T.N. Popova, A.A. Agarkov, A.N. Verevkin

**Affiliations:** Voronezh State University, Universitetskaya pl., 1, Voronezh, Russia, 394006

**Keywords:** type 2 diabetes mellitusXXXX, biochemiluminescence, aconitate hydratase, citrate, caspase, epifamin

## Abstract

The effect of epifamin on free radical processes, the activity of caspase-1 and -3, aconitate hydratase and citrate content in rat’s liver at experimentally induced type 2 diabetes mellitus (T2DM) was studied. The action of epifamin at T2DM leads to a decrease in biochemiluminescence parameters, characterizing the intensity of free radical processes, and changes in aconitase activity and citrate level towards the control. Activities of caspase-1 and caspase-3 in the tissue decreased by a factor of 2.4 and 1.6 in comparison with the levels at the disease. Apparently, epifamin-mediated correction of the level of melatonin, providing a significant antioxidant effect, promotes positive action on the free radical homeostasis.

## INTRODUCTION

Diabetes mellitus is one of the existing socially significant diseases and remains a challenge both for fundamental medicine and for public health services. T2DM is responsible for over 90% of all of the cases of this pathology.

Free-radical oxidation of biomolecules plays a significant role in the pathogenesis of T2DM complications. The rate of free radical formation at T2DM depends on the rate of protein glycosylation and, therefore, on the degree of hyperglycemia, as well [1]. One of the reasons behind the enhancement of free-radical processes at T2DM may be associated with the activation of the polyol pathway, whose function is geared towards converting glucose to sorbitol with the participation of aldose reductase. Free radicals at hyperglycemia can also be formed via glucose autoxidation as the final glycosylation products are formed; in turn, these products participate in angiopathy pathogenesis, increase ischemia, and intensify the free-radical processes in tissues at T2DM [1]. Increased glycosylation of hemoglobin causes secondary tissue hypoxia [2].

The disruption of redox homeostasis at T2DM may be the reason behind the induced apoptosis, programmed cell death, which is characterized by the activation of a cascade of intracellular cysteine proteases (known as caspases). Caspases are a family of evolutionarily conserved proteases that are capable of cleaving proteins at specific sites after asparaginic acid residues [3]. In particular, caspase-3 participates in the proteolysis of the inhibitor of the DNase responsible for DNA fragmentation (CAD). Furthermore, caspases induce the hydrolysis of the lamin proteins that reinforce the nuclear membrane, resulting in chromatin condensation. They participate in the disintegration of the proteins that maintain the structural and functional status of the cytoskeleton; in the inactivation and disruption of the regulation of the proteins involved in DNA reparation, mRN A splicing, and DNA replication. Caspase-3 (cpp32) is the key enzyme in the caspase family; assessing its activity is one of the main methods used to determine the level of apoptosis in tissue [4]. Activity of caspase-1 (ICE ), which belongs to the group of cytokines (caspase activators), is another important parameter characterizing the apoptotic process.

Aconitate hydratase (AH), which plays the key role in the regulation of citrate accumulation, is known to be one of the sensitive targets of free radicals [5]. It was demonstrated that the regulation of AH activity is significantly changed by the activation of freeradical oxidation, resulting in the suppression of enzymatic activity and accumulation of citrate, which is a low-molecular-weight antioxidant due to its chelating properties for Fe^2+^ [6]. Fe^2+^ ions are known to exhibit a pro-oxidant activity, since they help produce a hydroxyl radical, one of the most aggressive and dangerous reactive oxygen species (ROS), via the Fenton and Haber–Weiss reactions [7].

The use of drugs that would reduce the intensity of free-radical processes in the organisms remains rather topical. Epifamin is a peptide bioregulator that exhibits tropicity for the epithalamic-epiphyseal area. It belongs to the family of cytomedins. In addition to having a positive effect on the immune system and normalizing the fat and carbohydrate metabolism, these peptides can also correct the endrogenous melatonin level [8, 9]. The antioxidant mechanism is one of the main biochemical mechanisms used by melatonin to impact cells. Melatonin is an active electron donor and an efficient scavenger of radicals (OH^•^, OOH, O_2_^•-^ , singlet oxygen, NO^•^, ONOO^•^P) [10]. Unlike most other intracellular antioxidants that localize primarily in certain cellular structures, the presence of melatonin and its antioxidant activity have been detected in all cellular structures, including the nucleus [11]. This fact attests to the universal nature of the antioxidant effect of melatonin and to the pronounced protective properties that ensure protection of DNA, proteins, and lipids against free radical damage.

This work was aimed at studying the effect of epifamin on the intensity of free-radical processes, activity of caspase-1 and 3 and aconitate hydratase, and on the citrate level in the liver of rats with experimental T2DM.

## EXPERIMENTAL

White male rats (*Rattus rattus *L.; 150–200 g) were used for the experiments. All the procedures were performed in compliance with the Guidelines for Humane Care and Use of Laboratory Animals and the sanitary rules for maintenance of experimental biological clinics (vivarium). T2DM was induced via intramuscular injection of protamine sulfate during 3 weeks at a dose of 10 mg/kg b.w. (0.5 ml of 0.9% NaCl) thrice daily [12].

The animals were divided into three groups: group 1 (*n *= 8) consisted of the control animals; group 2 (*n *= 8) included the animals with T2DM; group 3 (*n *= 8) included the animals with T2DM, which intraperitoneally received the epifamin solution (in 1 ml of 0.9% NaCl solution) thrice daily at a dose of 2.5 mg/kg on day 15, 17, and 19. Three weeks after the induction of T2DM had started, the narcotized animals in all the experimental groups were euthanized and used for further analysis. The liver was removed as follows: anesthetized rats were subjected to laparotomy; a ligature was placed below the portal vein; the vein was incised, and a cannula was inserted 10 mm below the sinus. The anterior vena cava was cut in the diaphragm area; the liver was perfused with an ice-cold isotonic solution at a rate of 5 ml/min for 5 min. A weighed portion of tissue was homogenized by grinding it with quartz sand in a porcelain mortar with a 4-fold volume of a cold medium. The medium consisted of 0.1 M Tris-HCl buffer (pH 7.8), 1 mM EDTA, and 1% β-mercaptoethanol. The homogenate was centrifuged at 10,000 *g *for 12 min. The supernatant fluid was used for further study.

The glucose level in the rat blood serum was determined by the glucose oxidase method using the GLUC OSE-12-VITAL reagent kit (OOO Vital Diagnostics, St. Petersburg, Russia). Blood samples were collected from the tail vein on days 15, 17, and 19 [12].

Serum was obtained by short-term centrifugation. Induced biochemiluminescence (BCL) was used to determine the intensity of free-radical processes [14]. The BCL kinetic curve was recorded on a BCL-07 biochemiluminometer with software during 30 s, and the following parameters were determined: light sum (*S*) corresponding to the area below the chemiluminescence curve; the maximum flash intensity (*I*_max_) – the maximum value on the biochemiluminescence curve characterizing the intensity of free-radical processes; and the slope of the tangent line to the BCL curve (tgα_2_), which characterizes the total antioxidant activity.

The medium used to determine the BCL intensity consisted of 0.4 ml of a 0.02 mM potassium phosphate buffer (pH 7.5), 0.4 ml of 0.01 mM FeSO_4_, and 0.2 ml of a 2% hydrogen peroxide solution (added immediately prior to the measurement). The analyzed material (0.1 ml) was introduced directly prior to the measurement before adding hydrogen peroxide.

Caspase-1 and 3 activities were determined using the Caspase 1 Assay Kit, Colorimetric and the Caspase 3 Assay Kit, Colorimetric, respectively (both kits were purchased from Sigma), on a Hitachi U1900 spectrophotometer with software. The colorimetric analysis of caspase activity is based on the hydrolysis of the peptide substrates acetyl-Tyr-Val-Ala-Asp*-p-*nitroanilide (Ac-YVAD-pNA) (for caspase-1) and acetyl-Asp-Glu- Val-Asp-*p-*nitroanilide (Ac-DEVD-pNA) (for caspase-3), yielding *p*-nitroaniline with the adsorption peak at 405 nm (molar extinction coefficient = 10.5). Caspase activity was expressed as picomoles of the product formed during 1 min calculated for 1 mg of the protein.

The AH activity was measured on a Hitachi U1900 spectrophotometer at 233 nm. The rate of citrate dehydration was assessed from the formation of a double bond in the *cis*-aconitate molecule. The AH activity was determined in a 50 mM Tris HCl buffer, pH 7.8, containing 0.15 mM citrate. A unit of enzyme activity (E) was defined as the amount of enzyme catalyzing the formation of 1 μmol of the reaction product during 1 min at 25°C.

The amount of citrate was determined using the Natelson technique [15]. This method is based on bromination of citrate in the presence of potassium permanganate yielding pentabromoacetone, which reacts with thiourea to produce a colored complex. The color intensity of this compound was measured spectrophotometrically at 430 nm on a Hitachi U1900 spectrophotometer. The calibration curve was used for calculations.

The total amount of protein was determined using the biuret test. The statistical significance of differences was assessed by Student’s *t*-test. Differences at *p *≤ 0.05 were regarded as statistically significant.

## RESULTS AND DISCUSSION

**Fig. 1:**
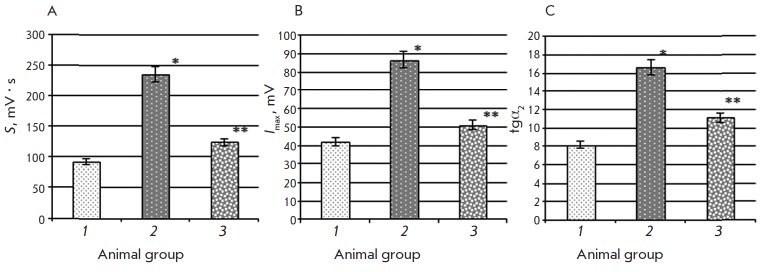
Biochemiluminescence parameters: Light sum (S), mV * c (A), maximum flash intensity (Imax), mV (B), the slope of the tangent line to the kinetic curve (tg α2) (C) in rat liver in the control group (1), animals with type 2 diabetes mellitus (2), and after introduction of epifamin into animals with pathology (3) Note. The differences are significant at p ≤ 0.05: * – compared to the control group, ** – compared to the group of rats with T2DM.

**Table:** Glucose concentration in rat blood serum in the experimental groups on days 15, 17, and 19 after the beginning of the experiment

Animal group	Glucose concentration, mM		
	day 15	day 17	day 19
1 (control)	5.00 ± 0.24	5.26 ± 0.23	5.5 ± 0.26
2	9.02 ± 0.41	9.72 ± 0.43	13.74 ± 0.64
3	8.18 ± 0.38	7.92 ± 0.36	7.71 ± 0.34

The introduction of protamine sulfate into experimental animals was found to increase the glucose level in blood serum. The use of epifamin as a protector reduced the hyperglycemia level in rats with experimentally induced T2DM: on day 19 after the experiment was started, the glucose blood level in animals with T2DM that received epifamin was lower 1.8-fold as compared to that in animals with T2DM that did not receive epifamin (Table). This can be attributed to the ability of epifamin to increase the melatonin level in the organism. It is a known fact that melatonin can stimulate glucose transport to skeletal muscles, thus activating the IRS-1/PI-3-kinase pathway and reducing the glucose concentration in the blood [16].

According to the resulting data, the light sum (*S*) and the maximum flash intensity (*I*_max_) in the liver of rats with T2DM were 2.6- and 2.1-fold higher than the same parameters in the control animals (Fig. 1 A, B), thus attesting to the fact that the intensity of free-radical oxidation increases. In accordance with the published data, the polyol pathway in which glucose is converted to sorbitol with the participation of aldose reductase takes place upon T2DM. Sorbitol dehydrogenase converts sorbitol to fructose, which is accompanied by an increase in the NADH/NAD^+^ ratio, similar to that during the development of tissue hypoxia. This condition has become known as “reductive stress” or “hyperglycemic pseudohypoxia” [2]. This condition may change the degree of reduction of the components of the electron transport chain, thus increasing the probability of ROS formation.

A 2.1-fold increase in tgα_2_ (BCL parameter characterizing the total antioxidant activity) was also detected in the liver of animals with T2DM as compared to that in the control group (Fig. 1 C). The introduction of epifamin into rats with T2DM reduced the *S *and *I*max values by 1.9 and 1.7 times, respectively (Fig. 1 A, B). The recorded decrease in the free-radical oxidation level may result from a manifestation of the antioxidant properties of melatonin, whose level can be controlled by epifamin. According to the published data, melatonin can interact with a number of reactive oxygen metabolites and neutralize the hydroxyl radical, one of the most active ROS in particular [10, 17].

Furthermore, the tgα_2_ values in animals with T2DM that received epifamin were 1.5-fold lower than those in animals with T2DM that did not receive the agent. This can be attributed to the decrease in the degree of mobilization of the antioxidant system due to inhibition of free-radical processes.

**Fig. 2:**
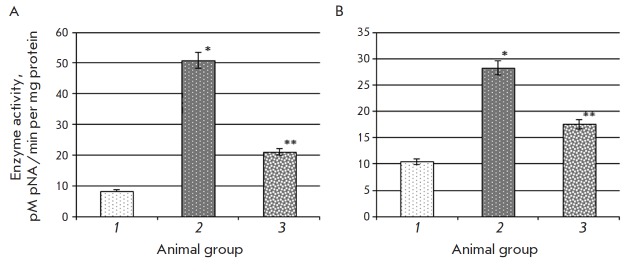
Activity of caspase-1 (A) and caspase- 3 (B) in rat liver in the control group (1), animals with type 2 diabetes mellitus (2), and after introduction of epifamin into animals with the pathology (3) Note. The differences are significant at p ≤ 0.05: * – compared to the control group, ** – compared to the group of rats with T2DM.

The specific activity of caspase-1 and 3 in the liver of rats with experimentally induced T2DM was found to increase 6.0- and 2.7-fold, respectively (*Fig. 2*). This is indicative of the enhancement of apoptotic processes in liver cells. An increased activity of caspase-3 in rat liver was also observed after the rats were exposed to carbon tetrachloride [18]. Introduction of epifamin into animals with T2DM reduced caspase-1 and -3 activities in liver 2.4- and 1.6-fold compared to the corresponding values in animals with T2DM that did not receive epifamin (*Fig. 2*).

**Fig. 3:**
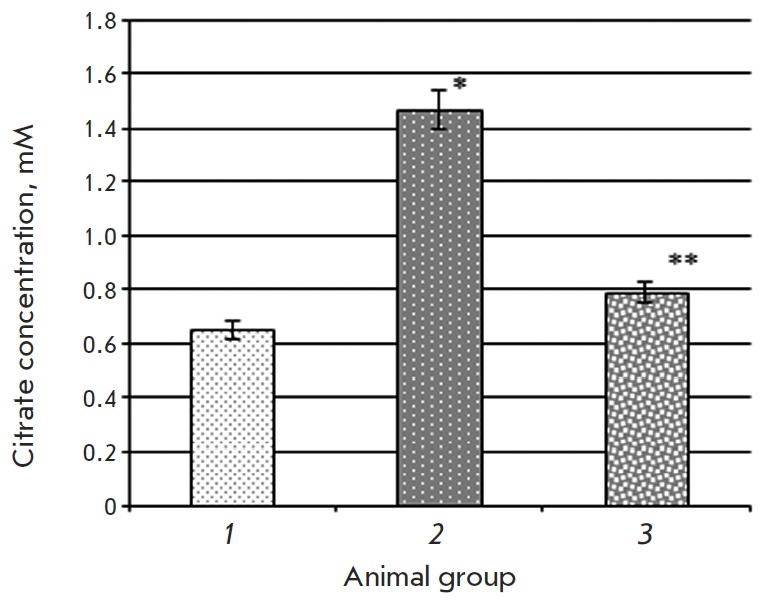
Citrate concentration in rat liver in the control group (1), animals with type 2 diabetes mellitus (2), and after introduction of epifamin into animals with the pathology (3) Note. The differences are significant at p ≤ 0.05: * – compared to the control group, ** – compared to the group of rats with T2DM.

**Fig. 4:**
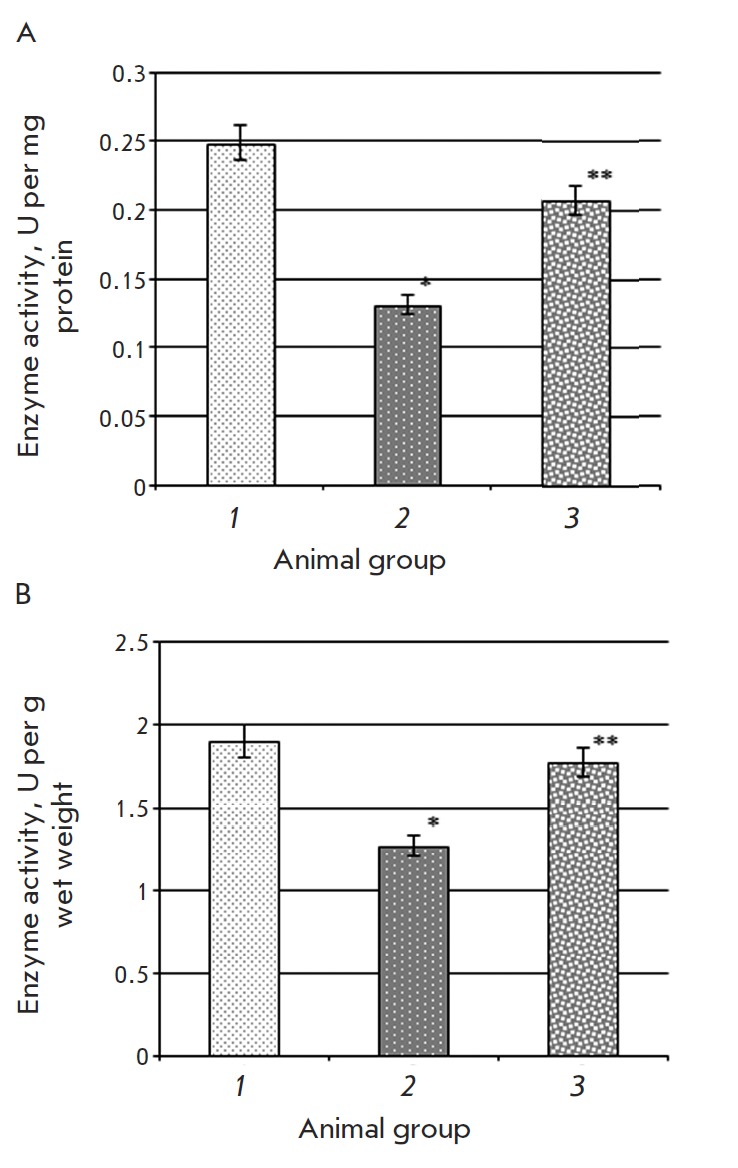
Aconitate hydratase activity in U per mg protein (A) and U per g wet weight (B) in rat liver in the control group (1), animals with type 2 diabetes mellitus (2), and after introduction of epifamin into animals with pathology (3) Note. The differences are significant at p ≤ 0.05: * – compared to the control group, ** – compared to the group of rats with T2DM.

Thus, the results demonstrate that epifamin reduces the level of apoptotic processes in the liver of rats with T2DM, which can presumably be attributed to the fact that the rate of free-radical processes decreases after the introduction of epifamin. It was shown that the citrate concentration in the liver of rats with T2DM increases 2.3-fold compared to the control values (Fig. 3). A 1.9-fold decrease in the specific activity of aconitase in the liver of animals with T2DM and a 1.5-fold decrease in activity (U/g dry weight) compared to the control were also observed (Fig. 4). It is well-known that the AH activity can be an oxidative stress marker, since the enzyme loses its activity under the action of ROS as the active site is modified and an iron atom is released from the iron–sulfur cluster [5]. The data regarding the changes in the AH activity and citrate concentration in animals with T2DM show agreement with the results of measurements of the BCL parameters, which attest to the fact that the intensity of free-radical oxidation increases under conditions of developing T2DM.

Introduction of epifamin into rats with T2DM reduced the citrate concentration in liver 1.9-fold (*Fig. 2*) and increased specific AH activity 1.6-fold (Fig. 4A) compared to these parameters in animals with T2DM that did not receive epifamin. The AH activity expressed as U/g wet weight of the liver also increased 1.4-fold as compared to the second experimental group (Fig. 4B). The changes in the tested parameters towards the control values after the animals with T2DM received epifamin apparently attest to the fact that the oxidative stress level fell, which resulted in reconstruction of the active site of AH and loss of citrate in the AH-catalyzed reaction.

## CONCLUSIONS

The data obtained demonstrate that epifamin has a positive regulating effect on free-radical homeostasis via a reduction in the intensity of oxidative stress in rats with induced T2DM. This fact is supported by the changes in the BCL indicators (*I*_max_ and *S*) that characterize the intensity of free-radical processes; in the tgα_2_ values that show the total antioxidant activity; in the activities of caspase-1 and caspase-3 indicating the rate of apoptotic processes; and in the AH activity and citrate concentration in rat liver under T2DM towards the normal values.

## Abbreviations

AH: aconitate hydratase
ROS: reactive oxygen species
BCL: biochemiluminescence
T2DM: type 2 diabetes mellitus.

## References

[1] Balabolkin M.I., Klebanova E.M. (2000). Problems of Endocrinology..

[2] Baynes J.W., Thorpe J.W. (1999). Diabetes..

[3] Kutsyy M.P., Kuznetsov E.A., Haziyev A.I. (1999). Biochemistry..

[4] Woo M., Hakem R., Soengas M.S., Duncan G.S., Shahinian A., Kägi D., Hakem A., McCurrach M., Khoo W., Kaufman S.A. (1998). Genes Dev..

[5] Gardner P.R., Nguyen D.M., White C.W. (1994). Proc. Natl. Acad. Sci. USA..

[6] Cadet E., Gadenne M., Capron D., Rochette J. (2005). Rev. Med. Interne..

[7] Kuhtina E.N., Glushchenko N.N. (1996). Biochemistry..

[8] Havinson V.H., Kvetnoy I.M., Yuzhakov V.V., Popuchiev V.V., Konovalov S.S. (2003). Peptidergicheskaya regulatsiya gomeostaza (Peptidergic regulation of homeostasis). St. Petersburg.: Scienes, 2003. 194 p..

[9] Anisimov V.N., Khavinson V.Kh. (2005). Aging interventions and therapies. Singapore: World Scientific.. World Scientific.

[10] Reiter R.J., Tan D.X., Osuna C., Gitto E. (2000). J. Biomed. Sci..

[11] Reiter R.J., Acuña-Castroviejo D., Tan D.X., Burkhardt S. (2001). Ann. N. Y. Acad. Sci..

[12] Ulyanov A.M., Tarasov Y.A. (2000). Vopr Med Khim..

[13] Bogomolov A.F., Lukyanov I.J., Gorbacheva L.R. (2005). Guidelines on the course of experimental physiology for students of biological department of biology and chemistry faculty. Ivanovo: Ivanovo State University, 2005. 43 p..

[14] Kuzmina E.I., Nelyubin A.S., Shchennikova M.K. (1983). Interuniversity collection: Biochemistry and Biophysics microorganisms..

[15] Afanasyev V.G., Zaitsev V.S., Wolfson T.I. (1973). Laboratornoe delo (Lab. Business)..

[16] Ha E., Yim S.V., Chung J.H., Yoon K.S., Kang I., Cho Y.H., Baik H.H. (2006). J. Pineal Res..

[17] Peschke E. (2008). J. Pineal Res..

[18] Lemza S.V., Azhunova T.A., Mondodoev A.G., Nikolaev S.M., Razuvaeva Y.G., Zandanov A.O. (2010). Bulletin VSNC SO RAMN (Bulletin of East-Siberian Scientific Center of the Siberian Branch of the Russian Academy of Medical Science)..

